# Pregnant Women Level of Satisfaction on Quality of Care in Reproductive and Child Health clinic at Huruma Designated District Hospital in Rombo District, Kilimanjaro Region, Tanzania.

**DOI:** 10.24248/eahrj.v4i1.621

**Published:** 2020-06-26

**Authors:** Simon Kamanda, John Majaliwa, Rashid Shehe, Florida Muro, Bernard Njau

**Affiliations:** a Institute of Public Health, Kilimanjaro Christian Medical University College, Moshi-Tanzania; b Kilimanjaro Christian Medical Centre, Mosh –Tanzania

## Abstract

**Background:**

The clients' level of satisfaction is an important measure in assessing the quality of health care services provided in health facilities, and is important in enhancing the utilisation of health care services.

**Objectives:**

This study aimed to determine pregnant women's level of satisfaction on the quality of care in the Reproductive and Child Health (RCH) clinic at Huruma Designated District Hospital, Rombo Kilimanjaro.

**Methodology:**

A cross-sectional study was conducted from May to June 2018 using the Donabediean model. Using systematic sampling, 270 pregnant women were selected to participate in the study. Data was collected using a pre-tested Service Quality(SERVQUAL) questionnaire. Descriptive statistics were performed using univariate and bivariate analysis, and one sample t-test to compare mean gap scores. The principal component analysis was employed to identify key items that measure the quality of care. A p-value of <.05 was considered statistically significant.

**Results:**

Overall, pregnant women's level of satisfaction on the quality of care in the Reproductive and Child Health clinic at Huruma DDH was 48.5%. The overall mean gap score (±SD) for the level of satisfaction was -0.53 (±1.69) signifying dissatisfaction with the quality of care. The overall level of satisfaction was associated with level of education (p<.001), occupation (p=.003), residence (p=.035). The levels of dissatisfaction in the 5 service dimensions were: empathy (-0.05), responsiveness (-0.09), assurance (-0.10), tangible (-0.13), and reliability (-0.17).

**Conclusion:**

Overall, pregnant women were dissatisfied with the quality of care provided. Pregnant women who are educated, being employed, and residing in Rombo were more likely to report dissatisfied with the quality of care. To improve the quality of care, lack of adequate staff and inadequate knowledge of the staff at RCH, and improvement in staff-clients interactions, and keeping scheduled appointments need to be improved.

## BACKGROUND

Globally, pregnancy and childbirth claim the lives of an estimated 303,000 women annually; with 50% of these deaths occurring in Africa.^1^ According to the 2015-2016 Tanzania Demographic and Health Survey and Malaria Indicator Survey, the maternal mortality rate in Tanzania was estimated at 386/100,000 live births, compared to 12/100,000 live births in developed countries.^1,2^ Besides, data shows that 98% of pregnant women attended Reproductive and Child Health (RCH) care at least once during their pregnancy, whereby 51% attained the recommended 4 visits. Furthermore, data shows that 64% received assisted-delivery at a health facility.^2^ Poor quality of RCH care is a key attribute of suboptimal institutional delivery in Tanzania and high maternal mortality rates.^2^

Clients' satisfaction is a measure of the quality of care of health system performance,^3^ and manifests itself as distribution, access, and utilisation of health services.^4^ Evidence from studies conducted in developed countries has shown that the overall level of satisfaction on the quality of RCH care among pregnant women differs from country to country, ranging from 81.5% in Belgium^5^ to 90% in Kazakhstan.^6^ In sub-Saharan Africa, similar differentials on the overall level of satisfaction on the quality of care were noted. The level of satisfaction ranged from 61.9% in Ethiopia,^7,8^ to 81.1% in Nigeria.^9^ To improve the quality of health care services, the Tanzanian government has made efforts through the Ministry of Health, Community Development, Gender, Elderly and Children (MoHCDEC), using different approaches to improve quality of care.^4^ These approaches include: supplying essential drugs for free and at low cost, provision of maternal and child health services for free in public health facilities, community education on maternal and childbirth, and increasing the number of health facilities.^10^ This study used the Donabedian model^11^ to determine pregnant women's level of satisfaction with the quality of care in the RCH clinic in the study area.

Briefly, the Donabedian model includes 3 key domains: structure, process, and outcome, which are interrelated in the assessment of the quality of care. The Donabedian model underscores the likelihood of a good structure to increase good process, which increases a good outcome (i.e, patients' satisfaction). The justification of selecting the Donabedian model in this study is based on substantial empirical evidence for its ability to generate information on the quality of care.^11^ Irrespective of the many studies conducted in Tanzania on patients' level of satisfaction with the quality of care, there is a paucity of data regarding pregnant women's level of satisfaction with RCH services in a Designated District Hospital^12^ in rural Tanzania. Therefore, this study aimed to determine pregnant women's level of satisfaction on the quality of care in an RCH clinic in rural Tanzania. Findings from this study will add knowledge to the literature by assessing how the Donabedian model might explain pregnant women's level of satisfaction on the quality of care, and provide recommendations for improvement of RCH services offered at the hospital.

## METHODS

### Study Design and Area

A cross-sectional study was conducted at Huruma DDH in the Rombo district from May to June 2018. Rombo is 1 out of 6 districts of the Kilimanjaro region. Huruma DDH is in the Rombo district council located in the north eastern part of the Kilimanjaro Region, Tanzania.

Huruma DDH serves Rombo District, which has an estimated population of 260,963 (Male=124,520(47.7%); Female=136,435(52.3%).^13^ Women of childbearing age(15 to 49 years) were 6,640. Rombo district has a total of 45 health facilities (37 dispensaries, 6 health centres, and 2 hospitals). Huruma DDH has a catchment area of 50,000 to 60,000, with 280 official bed capacity. As a DDH, it receives referred patients from other facilities within the catchment area and neighbouring Kenya. During the time of the study, approximately, 40 to 50 pregnant women per day attend the RCH clinic, which runs 5 days a week.

### Study Population

The study population comprised all pregnant women who attended the RCH clinic at Huruma DDH during the study period and consented to participate in the study. Participants who were mentally handicapped, re-attendances, and those with serious ill conditions were excluded.

### Sample Size and Sampling

A single population proportion sample size determination formula was used to calculate the sample size. Based on a study conducted by Olomi et al. (2017), the patients' level of satisfaction in the Kilimanjaro region was 20%^14^ and this was taken to calculate the sample size. A margin error of 5%, non-response rate of 10% and the desired level of Confidence Interval at 95% were included in the formula as follows: N=Z^2^xPx(100-P)/E^2^ where by N= Estimated Sample Size, Z=Standard Normal Deviation of 1,96^2^ corresponding to 95% Confidence Interval (CI), P = Proportion of outcome under study and E= Marginal Error at 5%.^15^

A minimum sample size of 245 was calculated, and adding 25 respondents (ie, 10% non-response rate), the actual estimated sample was 270. A systematic sampling based on the projected daily attendance at the RCH clinic and a list of attending clients obtained from the clinic register book was used to select participants in the survey. To determine the first interviewee, a simple random technique was employed by drawing a piece of paper written YES among 4 pieces of paper in a box placed at the registry section. A systematic sampling technique was employed to obtain the rest of the interviewees who met the inclusion criteria.

A formula N/n was used to obtain the sampling interval where by, N= the total number of clients attending at the RCH clinic per day and n= the estimated sample size.^15^ The RCH clinic at Huruma DDH runs daily, which gave a total of 28 days for data collection in 4 weeks but only weekdays were considered which gave a total of 20 days. Number of clients who had to be interviewed per day = Total number of clients for the study/Number of days. The number of clients who had to be interviewed per day=270/20=13.5,~14. Therefore, the number of respondents who had to be interviewed per session for the first 19 days comprised of 14 respondents but 4 respondents on the last day of the interview to accomplish a total of 270 study participants.

Anonymously, structured SERVQUAL questionnaire was adapted and then adopted to address the study objectives.^16^ The SERVQUAL questionnaire comprises 5 service dimensions (tangibles, reliability, responsiveness, assurance, and empathy) to determine clients' level of satisfaction on the quality of care. There are 2 categories of questions in assessing clients' level of satisfaction with regard to SERVQUAL questionnaire: 1) Expectation questions and 2) Perception questions.

The questionnaire was developed in English with back-and-forth translation to Kiswahili, the local language in Tanzania. The SERVQUAL questionnaire in Kiswahili was then piloted with a convenient sample of n=30(pregnant women) for validity and reliability. Based on the pilot testing, minor adjustments were done. Trained research assistants with previous experience in quality of care research conducted the data collection process.

### Study Variables

The independent variables in this study included: socio-demographic characteristics (age, religion, marital status, level of education, occupation, and residence). Pregnant women's level of satisfaction was the dependent variable in this study and was assessed by asking the level to which they were satisfied with the quality of care by looking at the gap between their expectations and perceptions using 4 point-Likert scale questions (rating points on the scale). (Table 1)

**TABLE 1.** Donabedian domains and expected response options measured in the survey

Domains	No. of items	Sample questions (response options)	Alpha
**Tangibles**	5	“I expect doctor of this RCH clinic to prescribe good drugs” (1= Strongly disagree to 4= Strongly agree)	.85
	5	“I am satisfied doctor of this RCH clinic has prescribed good drugs” (1= Strongly disagree to 4= Strongly agree).	
**Reliability**	4	“I expect staff of an excellent RCH clinic to have good communication and information skills” (1= Strongly disagree to 4= Strongly agree).	.78
	4	“I am satisfied staff of this RCH clinic have good communication and information skills” (1= Strongly disagree to 4= Strongly agree).	
**Responsiveness**	7	“I expect staff of an excellent RCH clinic to provide prompt service to clients” (1= Strongly disagree to 4= Strongly agree)	.81
	7	“I am satisfied staff of this RCH clinic offer prompt services” (1= Strongly disagree to 4= Strongly agree).	
**Assurance**	5	“I expect laboratory results of the excellent RCH clinic will be timely availed”. (1= Strongly disagree to 4= Strongly agree)	.76
	5	“I am satisfied laboratory results of this RCH Clinic are timely availed” (1= Strongly disagree to 4= Strongly agree).	
**Empathy**	5	“I expect staff of an excellent RCH clinic to listen to clients adequately”. (1= Strongly disagree to 4= Strongly agree).	.84
	5	“I am satisfied staff of this RCH Clinic listen to me adequately” (1= Strongly disagree to 4= Strongly agree).	

a Example of expectation questions

b Example of perception questions

### Data Management and Analysis

Data was edited, cleaned, coded, entered, and analysed using Statistical Package for Social Sciences (SPSS) version 20.1 (SPSS for Windows; SPSS, Chicago, IL, USA). Descriptive statistics such as frequency, percentages, the measure of central tendency (dispersion) for continuous variables were computed. The chi-square and p-value were used to assess the level of significance and strength of association in categorical variables. To calculate the mean gap score of the client's level of satisfaction, the following procedures were used: A total score (in %) was calculated for each domain (e.g. responsiveness) for both expectation and perception questions. Subtracting perception score from expectation score derived the total gap score. A one-sample t-test was used to compare means of continuous variables (client's expectations versus perceptions). The gap implies the level of clients' satisfaction with the quality of care. Quality of care is deemed indifferent or sufficient when the client's level of satisfaction is equal or greater than the expected level of service or vice versa.^16^

Also, Principal Component Analysis (PCA) was employed to identify the subgroups of the SERVIQUAL items forming sub-scales. Before performing PCA, the suitability of data was assessed. A Correlation Coefficient was set at a cut off point of .3 or above. The Kaiser-Meyer-Oklin value-which was used to assess sampling adequacy was set at a cut-off point of .6.

Bartlett's test of sphericity was used to support the factorability of the correlation matrix. Besides, a Catell's scree test, and an eigenvalue of over 1.0 which represents the amount of the total variance explained by a factor, were used to inspect the plotting of each eigen value of the factors to find a point at which the shape of the curve changes direction and becomes horizontal.

All factors above the break in the plot and with eigenvalues of over 1.0 were retained for further analysis. Finally, further analysis was done using the Varimax method, to try to minimize the number of variables with high loadings on each factor.

This research adhered to the STROBE guidelines for cross-sectional studies.^17^

### Ethical Consideration

Ethics approval was obtained from Kilimanjaro Christian Medical University College Research Ethics and Review Committee (CRERC) with ethical clearance number 569. Permission to conduct the study was sought from the Huruma DDH administration. Both written and verbal consent was obtained from eligible respondents at RCH clinic after they have been explained about the study objectives. Respondents were informed that their participation was voluntary, and they can withdraw at any stage of the study. Respondents were assured that their withdraw from the study will not affect their RCH care. To ensure confidentiality and privacy, respondent identification numbers instead of their names were used. Also, all interviews were conducted in a private place.

## RESULTS

A total of 270 female participants were included with a response rate of 100%. The mean (±SD) age of the participants was 26(±5.12) years. More than two-thirds of 173(64.0%) were aged between 21 and 29 years. The majority, 238(88.1%) were Christians, 207(76.7%) were married, 133(49.3%) had secondary school education and 240(88.9%) reside in Rombo. (Table 2)

**TABLE 2.** Socio-Demographic Characteristics of Study Participants (N=270)

Characteristic	Frequency	Percentage
**Mean age (±SD) Years**		
26.0(±5.12)	270	100
**Age groups**		
≤20	35	13.0
21-29	173	64.0
≥30	62	23.0
**Religion**		
Christian	238	88.1
Muslim	32	11.9
**Marital Status**		
Married	207	76.7
Single	63	23.3
**Level of Education**		
Primary education	79	29.2
Secondary education	133	49.3
College/University	58	21.5
**Occupation**		
Employed	83	30.7
Self employed	95	35.2
Unemployed	92	34.1
**Residence**		
Rombo	240	88.9
Other places	30	11.1

### Overall Pregnant Women Level of Satisfaction with the Quality of Care

The overall pregnant women's level of satisfaction with the quality of care in the RCH clinic at Huruma DDH was 48.5% (n=131/270). The overall mean gap score (±SD) for pregnant women's level of satisfaction was relatively small -0.53(±1.69) signifying dissatisfaction among pregnant women in RCH service provision. The mean expectation score was 17.56 while the mean perception score was 17.03. Therefore, the mean gap score (mean perception score-mean expectation score) was -0.53 for all the service dimensions assessed.

### Socio-Demographic Characteristics and Overall Level of Satisfaction with the Quality of Care

In this study, 3 socio-demographic characteristics, namely; the level of education, occupation, and residence were associated with the overall level of satisfaction of the quality of care. Preg nant women with secondary education had higher proportions of being dissatisfied with the quality of care compared with pregnant women with primary education (69.2% Vs 27.8%; p=<.001). Pregnant women who were employed were dissatisfied with the quality of care compared with unemployed pregnant women (66.3% Vs 41.3%; p=.003). Dissatisfaction with the quality of care was highest among pregnant women residing in Rombo compared to those residing outside the district (53.8% Vs 33.3%;p=.035).Table 3

**TABLE 3.** Socio-Demographic Characteristics of Study Participants and Patients' Level of Satisfaction (N=270)

Characteristic	Frequency (%)	Satisfied (%)	Dissatisfied (%)	Chi-square (p-value)
**Age Groups**				6.532(.083)
≤20	35 (13.0)	23 (65.7)	12 (34.3)	
21-29	173 (64.0)	84 (48.6)	89 (51.4)	
≥30	62 (23.0)	24 (38.7)	38 (61.3)	
**Religion**				1.765 (.184)
Christian	238 (88.1)	119 (50.0)	119 (50.0)	
Islam	32 (11.9)	12 (37.5)	20 (62.5)	
**Marital Status**				977(.323)
Married	207 (76.7)	97 (46.9)	110 (53.1)	
Single	63 (23.3)	34 (54.0)	29 (46.0)	
**Level of Education**				35.961 (.000)*
Primary education	79 (29.2)	57 (72.2)	22 (27.8)	
Secondary education	133 (49.3)	41 (30.8)	92 (69.2)	
College/University	58 (21.5)	33 (56.9)	25 (43.1)	
**Occupation**				11.433 (.003)*
Employed	83 (30.7)	28 (33.7)	55 (66.3)	
Self employed	95 (35.2)	49 (51.6)	46 (48.4)	
Unemployed	92 (34.1)	54 (58.7)	38 (41.3)	
**Residence**				4.450 (.035)*
Rombo	240 (88.9)	111 (46.2)	129 (53.8)	
Other places	30 (11.1)	20 (66.7)	10 (33.3)	

* p-value less than .05

The 54 items of the SERVIQUAL scale were subjected to Principal Component Analysis (PCA). Before conducting PCA, the suitability of the data for factor analysis was assessed. Inspection of the correlation matrix revealed the presence of many coefficients of .3 and above. The Kaiser-Meyer-Olkin value was .93, exceeding the cut–off point of .6, and Bartlett's test of Sphericity was statistically significant (p=.000). Principal Component Analysis revealed the presence of 10 components with eigenvalues exceeding 1, explaining 42.4%, 6.9%, 4.9%, 4.1%, 3.7%, 3.3%, 2.6%, 2.3%, 2.0%, and 1.9%. These 10 components explained a total of 74% of the variance.

Using Catell's scree test, 2 components above the breakpoint on the scree plots of factors for both expectation and perception scale were retained for further analysis. Further analysis using the Varimox method revealed strong loading eight (8) factors with both components. The 8-factor solution explained a total of 49.4% of the variance, with component 1 contributing 26.7%, and component 2 contributing 22.7%. The 8 items included: empathy (4 items), assurance (2 items), responsiveness (1 item), and reliability (1 item). Internal reliability of the items as indicated by Cronbach's coefficients of .95 for expectation subscale and .96 for perception subscale. (Table 4)

**TABLE 4.** Varimax Rotation of Two Factor Solution for SERVQUAL Items

Items	Component 1 Expectation score	Component 2 Perception score
1. RCH staff paid attention to my individual medical concerns.	.64	.81
2. RCH staff are polite, comforting and encouraging to me when faces with medical problems.	.57	.80
3. RCH staffs have built good rapport and ready to offer medical assistance.	.65	.79
4. RCH has adequate staff to attend pregnant women.	.42	.76
5. RCH staffs showed compassionate to me	.48	.74
6. RCH staffs have adequate knowledge to answer my questions.	.61	.74
7. RCH staffs showed willingness to help pregnant women.	.69	.70
8. RCH staff kept my scheduled appointments.	.57	.69
% of variance explained	26.7%	27.7%
Cronbach's alpha coefficients	.95	.96

## DISCUSSION

This study was conducted to determine pregnant women's level of satisfaction with the quality of care provided at the RCH clinic in Huruma DDH, in the Rombo district. In general, the overall pregnant women's level of satisfaction was 48.5% with a relatively small overall mean gap score signifying overall dissatisfaction with the quality of care. This finding is contrary to studies done in Oman (59%),^18^, Ethiopia (60.4%),^8^ Belgium (81.5 %),^5^ and Nigeria (81.1% to 90%).^9,19^ The probable explanation on the observed differentials could be because of variations in the study areas, the study populations, different level of expectations among patients, or actual lower levels of perceived quality of services provided.^14^

In this study, we found that some pregnant women's socio-demographic characteristics were important in determining their level of satisfaction. 3 socio-demographic characteristics namely; the level of education, occupation, and residence were important determinants for pregnant women's level of satisfaction with the quality of care. These findings are in line with studies conducted in Malaysia,^20^ and Ibadan, Nigeria.^9^ However, this finding is contrary to a study conducted in Ghana.^21^

For instance, dissatisfaction with the quality of care among employed pregnant women, in our study could be explained by the fact that women who are employed have higher expectations about the care they will receive. An alternative explanation could be their financial capability to pay for care in different health facilities irrespective of the charges or costs of treatment.^5^

Our study also found that pregnant women's level of satisfaction significantly differs by place of residence. Most pregnant women from Rombo were dissatisfied with the quality of care than those coming from outside the district. The most plausible explanation for the current observation could be the familiarity with health care providers at the RCH clinic. An alternative explanation could be the fact that Huruma DDH is the only district hospital, hence pregnant women lack alternative hospitals to compare the quality of care. Of all the 5dimensions used to assess pregnant women's level of satisfaction in this study, participants were least satisfied with the empathy dimension, followed by assurance, reliability, tangible, and responsiveness. The gap scores, which represent the discrepancy between pregnant women's expectation and perception for all 5 dimensions on quality of care, were negative.

According to the Donabedian model, empathy is used to assess the process domain.^11^ Empathy items which explained most of the dissatisfaction on quality of care were failure to pay attention to individuals' medical concerns, failure to show compassion, lack of politeness, comforting, and encouragement to pregnant women when facing medical problems, and inability to build a good rapport and lack of readiness to offer medical assistance.

The observed level of dissatisfaction of pregnant women on RCH staff's inability to build a good rapport and readiness to offer medical assistance, for example, underscores the importance of establishing staff-client relationships, to enhance the quality of care at RCH clinic. Existing evidence shows that there is a strong link between the client's satisfaction and retention in the health care delivery system.^22^

The assurance dimension in the Donabedian model is used to assess the structure domain.^11^ In this study, assurance items, which explained most of the dissatisfaction on quality of care, were lack of adequate staff to attend pregnant women, and inadequate knowledge of staff to respond to pregnant women's questions. For instance, the observed level of dissatisfaction of respondents on inadequate staff at the RCH clinic substantiates the major challenge of limited human resources for health sector in the Tanzanian health care delivery system.^23,24^ This observation calls for increased efforts by the government of Tanzania, through the MoHCDGEC to address the issue of the lack of health care personnel at all levels of the health care delivery system, including the RCH clinic, at Huruma DDH.^24^

However, this finding should be interpreted with caution, because the study did not assess the actual number of health care personnel attending pregnant women at the RCH clinic. Our finding is contrary to a study done in Egypt using the SERVQUAL tool, whereby pregnant women were more satisfied with adequate staff in the RCH clinic.^25^

The responsiveness dimension is used to assess the process domain in the Donabedian model.^11^ Of all the 7 items used to assess responsiveness, pregnant women were dissatisfied with the lack of RCH staff willingness to help pregnant women, when medical help is needed. Considering that dissatisfaction can be a major de-motivating factor in the use of RCH care, enhancing health providers' willingness to help pregnant women could result in a better relationship with the clients, ultimately improving the quality of care. Finally, the reliability dimension is used to assess the process domain as per the Donabedian model.^11^ Of all the 4 items used to assess reliability, respondents were least satisfied with the inability of RCH staff to keep their scheduled appointments. Existing evidence suggests that continuity in seeing the same health care provider during scheduled appointments could result in more regular consultations.^5,22^ It is imperative for the hospital management at Huruma DDH to make sure that scheduled appointments are adhered to by RCH staff for the betterment of the quality of care at the RCH clinic.

### Limitations and Strengths of the Study

This study has some limitations. The study design used cross-sectional which measures only the outcome of interest but is unable to identify the causality of the outcome of interest. Furthermore, the study focused on interviewing only pregnant women but excluded the health care providers, which might affect the study findings in terms of assessing the client-provider interactions. Further research is needed among pregnant women and health care providers in similar settings which will assess the effect of client-provider interactions. Selection bias could not be avoided completely in this study, because we only recruited pregnant women who attended the RCH clinic, and were unable to reach pregnant women without any access to RCH care. Also, selection bias is a possibility since the study excluded pregnant women younger than 18 years old due to ethical reasons. Although this study did not gather information on the number of pregnancies, another limitation that should be put into consideration is that women with few numbers of pregnancies may have limited experiences with RCH care compared to women with many numbers of pregnancies.

The SERVQUAL questionnaire added strength to this study because it is a standardised universal tool used to measure the quality of care, which has been used in different settings. Although several studies supported the validity and reliability of the SERVQUAL questionnaire, it should be tested with more demographical and culturally diverse samples.

## CONCLUSION AND RECOMMENDATIONS

This study provides useful insights for enhancing the quality of care for pregnant women attending RCH clinics. The results showed that generally pregnant women were dissatisfied with the quality of care, evidenced by negative gaps between perceptions and expectations scores, which imply that pregnant women's expectations were not met. Pregnant women with secondary education, being employed and residing in Rombo were more likely to report dissatisfaction with the quality of care. To improve the quality of care, lack of adequate staff and inadequate knowledge of the staff at RCH, and improvement in staff-clients interactions, and keeping scheduled appointments need to be improved.
